# Incidence of second primary cancers in patients with retinoblastoma: a systematic review and meta-analysis

**DOI:** 10.3389/fonc.2024.1372548

**Published:** 2024-03-28

**Authors:** Jinfeng Sun, Xiuli Gu, Liangjun Wang

**Affiliations:** ^1^ Department of Ophthalmology, Yantaishan Hospital Affiliated to Binzhou Medical University, Yantai, China; ^2^ Medical Services Division, Yantaishan Hospital Affiliated to Binzhou Medical University, Yantai, China

**Keywords:** retinoblastoma, second primary cancers, hereditary, meta-analysis, systematic review

## Abstract

**Introduction:**

This systematic review and meta-analysis aimed to examine the risk of second primary cancers (SPCs) among retinoblastoma (Rb) patients, both hereditary and nonhereditary. Previous studies have reported on the long-term risk of SPCs in these patient populations, but a comprehensive synthesis of the existing evidence is lacking.

**Methods:**

A systematic search was conducted in PubMed, EMBASE, and Cochrane Library from inception to 12 March 2023, supplemented by manual screening. Eligible studies were identified, and data were extracted. The primary outcome measure was the standardized incidence ratios (SIRs) of SPCs in Rb patients. Summary estimates were calculated using random or fixed effects models. The quality of included studies was assessed using the Newcastle-Ottawa Scale.

**Results:**

Ten studies, including nine high-quality studies, were included in this review. The summary estimate of SIR for SPCs among hereditary Rb patients was 17.55 (95% CI=13.10-23.51), while the pooled estimate of SIR for SPCs among nonhereditary Rb patients was 1.36 (95% CI=0.90-2.04). Significant differences in SIRs for different SPC types were observed (P=0.028), including nasal cavity tumor (SIR=591.06, 95% CI=162.79-2146.01), bone tumor (SIR=442.91, 95% CI=191.63-1023.68), soft tissue sarcoma (SIR=202.93, 95% CI=114.10-360.93), CNS (SIR=12.84, 95% CI=8.80-18.74), and female breast cancer (SIR=3.68, 95% CI=2.52-5.37). Chemotherapy and radiation therapy were associated with an increased risk of SPCs among hereditary Rb patients.

**Discussion:**

The findings of this review indicate that hereditary Rb patients have a significantly elevated risk of developing SPCs, whereas nonhereditary Rb patients do not show the same risk. Furthermore, significant differences were observed in the SIRs of different SPC types. Treatment techniques, specifically chemotherapy and radiation therapy, were associated with an increased risk of SPCs among hereditary Rb patients. These findings highlight the importance of radiation protection for Rb patients and the need for further research and tailored management strategies for this high-risk population.

## Introduction

Retinoblastoma (Rb) is the most common primary intraocular cancer in children and a relatively rare disease, occurring in one out of every 16,000-18,000 live births globally (1, 2). Around 95% of Rb were diagnosed before the age of 5 years and it accounts for 2% of all pediatric cancers (2, 3). Rb presents in heritable and nonheritable forms. Hereditary Rb is characterized by a germline mutation of the *RB1* gene, while nonhereditary Rb is developed due to two random mutations in the *RB1* gene in one cell of the retina, and it is also known as “‘sporadic’ or ‘somatic’ Rb (4). Early detection and intervention are crucial to the successful management of Rb. Rb is considered a curable disease with a near 100% disease-free survival rate in high-income countries, but the global survival rate is merely 30% (5). Systematic chemotherapy is considered the first line therapy for Rb treatment, and intra-arterial chemotherapy (IAC) is a targeted therapy which is more commonly adopted in developed countries (4, 6). Other therapies, including cryotherapy, transpupillary thermotherapy (TTT), external beam radiotherapy (EBRT), and plaque brachytherapy, are used in conjunction with systematic chemotherapy or IAC for tumor control (6).

Clinical evidence has suggested that individuals with hereditary Rb have a higher chance of developing second primary cancers (SPCs) than those with nonhereditary Rb (7–10). Common SPCs in Rb include osteosarcoma, soft tissue sarcoma, melanoma and epithelial cancers, which are associated with excess mortality (11). Significantly reduced risks among nonhereditary survivors, in comparison to hereditary survivors, is likely due to variations in both the inherent genetic vulnerability to SPCs and differences in treatment approaches (12). Some studies showed that those who have treated with radiation therapy (RT) have a higher risk of SPCs, while other studies have conflicting results regarding the risk of chemotherapy (13–15). Studies have demonstrated that chemotherapy is an independent risk factor for SPCs among Rb survivors, especially sarcoma (9, 10), while other studies found that systemic chemotherapy plays a protective role in the prevention of SPCs and metastases (6, 16).

Standardized incidence ratio (SIR) is generally used to compare cancer risk after retinoblastoma relative to the general population. Increasingly, studies have reported the SIR of SPCs in hereditary and nonhereditary Rb patients. For instance, Schonfeld et al. (2021) found that among hereditary and nonhereditary Rb survivors in New York and Boston, hereditary survivors had statistically significantly increased SPC risk (SIR = 11.9), and significantly increased risks were observed for sarcomas, nasal cavity tumors, central nervous system (CNS), and breast cancer (12). In contrast, SPC risk was not increased after nonhereditary Rb (SIR=0.8). Villanueva et al. (2022) reported that compared to the general Argentinean population, the risk of SPCs was 48-fold higher in hereditary Rb survivors (SIR=48.5) and four-fold in nonhereditary survivors (SIR=4.1) (15). Additionally, RT and chemotherapy further increased the risk of SPCs in Rb survivors (15). Nonetheless, no systematic review has been conducted to compile the evidence on risk of SPCs in hereditary and nonhereditary Rb, and the risks of different SPC types and treatment modalities are still unclear.

A systematic review and meta-analysis is needed to synthesize the existing evidence on the risk of SPCs in Rb patients. The findings would help identify the high population and inform guidelines and practices for disease surveillance and prevention in this population. Advanced disease surveillance would improve Rb patients’ survivorship and reduce mortality related to SPCs. To the best of our knowledge, there has not been a comprehensive systematic review or meta-analysis of the risk of SPCs among Rb patients. Therefore, the present study aimed to conduct a meta-analysis to examine the risk of SPCs among hereditary and nonhereditary Rb patients.

## Methods

### Search strategy and data sources

This meta-analysis was performed and reported according to the Cochrane Handbook for Systematic Reviews and the Preferred Reporting Items for Systematic Reviews and Meta-Analyses (PRISMA) criteria (17, 18).

A search of the following electronic databases from inception until 12 March 2023 was conducted: PubMed, EMBASE, Web of Science, and Cochrane Library. The following MeSH search terms were used: (“Retinoblastoma”) AND (“Neoplasm, Second Primary”). A search filter was applied to limit results to the English language, and we also retrieved articles by manual screening.

### Inclusion and exclusion criteria

The inclusion criteria include: (1) patients were diagnosed with retinoblastoma (hereditary or nonhereditary) (Participants); (2) patients had follow-up for the new primary cancer (the incidence of new cancers was calculated by the standardized incidence ratios (SIRs)) (Outcomes); (3) retrospective or prospective cohort studies were included (Study design). Reviews, conference abstracts, case reports, and animal trials were excluded from this study.

### Data extraction

Data from the eligible studies were extracted by two authors independently. The extracted data included authors, publication year, study location, studied years, type of retinoblastoma, types of SPC, and measured outcome (SIRs) with 95% CI. Any discrepancies were resolved through a consensus discussion with all the authors.

### Study quality assessment

The quality of the articles included was assessed using the Newcastle-Ottawa Scale (NOS) (19). The NOS contains three main dimensions: selection, comparability, outcome (cohort study), and exposure (case-control study). The quality score ranged from 0 to 9, and a higher score indicates better methodological quality. The NOS score < 7 is defined as low quality, and a score ≥ 7 as high quality. Where scores differed, discrepancies were resolved by discussions with all the authors.

### Statistical analysis

Stata V.14.0 (StataCorp LP) was used to assess the SIRs of the SPCs among Rb patients. The outcomes were shown as SIRs with their 95% CI. The Cochrane *Q* and *I^2^* statistics were used to test the heterogeneity among all studies. Heterogeneity was estimated using *I^2^* statistics; *I^2^* <25 was regarded as the absence of heterogeneity, 25% to 50% was considered a moderate level of heterogeneity, and *I^2^* >50% was considered substantial. Where *I^2^* was >50%, a random-effect or fixed-effect model was adopted to calculate the pooled SIRs and 95% CI. Subgroup analyses were conducted according to types of SPC (i.e., soft tissue sarcoma, bone tumor, central nervous system (CNS) cancer, nasal cavity cancer, and female breast cancer) and treatments (radiation, chemotherapy, and radiation plus chemotherapy). A sensitivity analysis was performed to test the robustness of the pooled results.

The funnel plot symmetry was used to check the potential of publication bias of the included studies. In meta-analysis, funnel plot is a useful graph to examine the existence of publication bias. Symmetrical funnel shapes indicate that publication bias is unlikely, but asymmetrical funnels indicate that publication bias may exist. However, some authors (20) have argued that the visual interpretation of funnel plots is too subjective and therefore not practical. Then Egger’s test was further used to more objectively test for its presence. Egger’s test uses a linear regression approach to interpret the asymmetric of funnel plots.

*P*<0.05 was considered statistically significant.

### Patient and public involvement

No patients were directly involved in this study.

## Results

### Study selection

**Figure 1:**
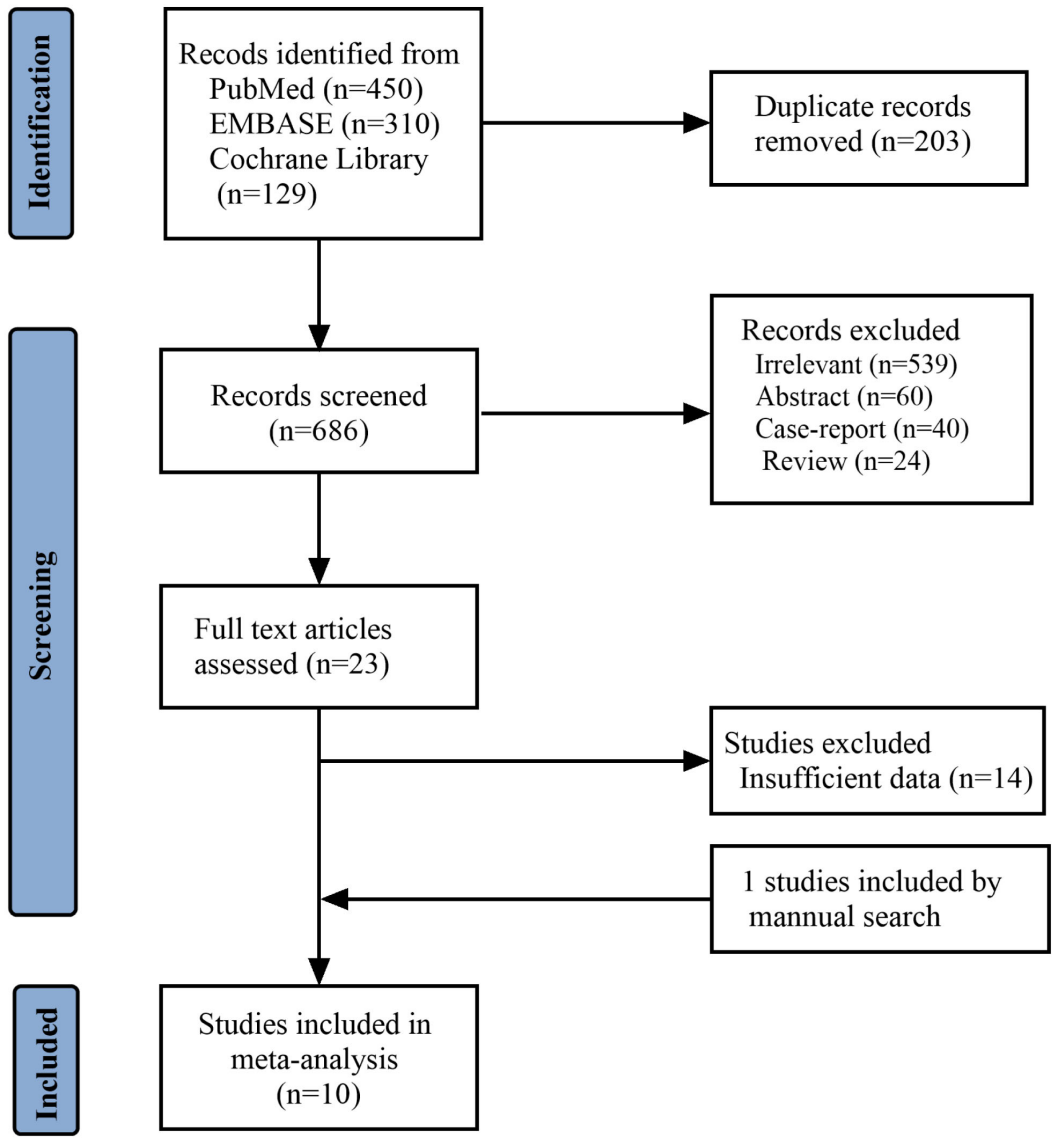
Flow-chart showing the article-screening process.

**Figure 1** reports the PRISMA flowchart of the study selection: A total of 1680 records were identified, of which 203 were duplicates. A total of 663 studies were excluded after screening the titles and abstracts, and 14 more studies were excluded due to insufficient reported data during full-text screening. One study was included through a manual search. Ten studies (7, 9, 12, 14, 15, 21–25) were included in the meta-analysis, with 10594 Rb patients.

### Study characteristics

All the included studies were retrospective cohort studies. Five studies were conducted in the United States, one in the United Kingdom, one in Germany, one in Denmark, one in the Netherlands, and one in Argentina; nine studies were conducted in developed countries and only one in a developing country. The sample sizes ranged from 323 to 2052 participants. Six studies involved hereditary and nonhereditary patients and four involved hereditary patients only. Study characteristics are presented in **Table 1**.

**Table 1:** The characteristics of the included study in our meta-analysis.

Study	Country	Participants	Study design	Study years	Type of Rb	NOS
Kleinerman et al., 2019 (22)	USA	952	Retrospective cohort	1914-2006	Hereditary	6
Gregersen et al., 2020 (14)	Denmark	323	Retrospective cohort	1943-2013	Hereditary and nonhereditary	7
Schonfeld et al., 2021 (12)	USA	2052	Retrospective cohort	1914-2006	Hereditary and nonhereditary	7
Temming et al., 2015 (23)	Germany	488	Retrospective cohort	NR	Hereditary	7
Kleinerman et al., 2005 (21)	USA	1601	Retrospective cohort	914-1984	Hereditary and nonhereditary	7
Marees et al., 2008 (24)	Netherlands	668	Retrospective cohort	1945-2005	Hereditary and nonhereditary	7
Kleinerman et al., 2007 (25)	USA	963	Retrospective cohort	1914-1984	Hereditary	7
Wong et al., 2014 (9)	USA	906	Retrospective cohort	1914-1996	Hereditary	7
MacCarthy et al., 2013 (7)	UK	1927	Retrospective cohort	1951-2004	Hereditary and nonhereditary	7
Villanueva et al., 2014	Argentina	714	Retrospective cohort	1987-2016	Hereditary and nonhereditary	7

### Study quality

Our study adopted the NOS to assess the quality of concerning studies. Therefore, the score of nine studies was equal to seven, considered high quality. The quality of one study could have been higher due to being below seven. The results of the study quality were shown in **Table 1**.

### The SIRs of SPCs in hereditary and nonhereditary Rb

**Figure 2:**
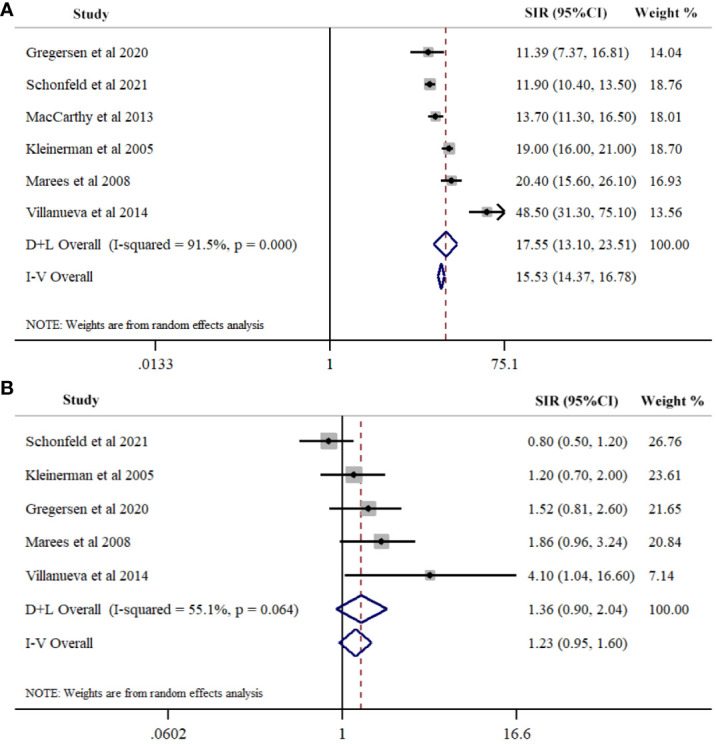
Meta-analysis of Risk for second primary cancers overall among hereditary **(A)** and nonhereditary **(B)** retinoblastoma patients. Overall effects were estimated using random effects model (D+L Overall).

We identified six studies reporting the SIRs of SPCs in hereditary Rb. The summary estimate of SIR for SPCs was 17.55 (95% CI=13.10-23.51; *I* ^2^ = 91.5%, *P*<0.001) (**Figure 2A**; **Table 2**). Five studies reported the SIRs of SPCs in nonhereditary Rb. The pooled estimate of SIR for SPCs was 1.36 (95% CI=0.90-2.04; *I* ^2^ = 55.1%, *P*<0.064) (**Figure 2B**; **Table 2**).

**Table 2:** The pooled standardized incidence ratios (SIRs) of second primary cancers in hereditary and nonhereditary retinoblastoma (Rb).

Stratified analysis	Number of studies	Number of patients	Pooled estimates (SIR with 95%CI)	Heterogeneity		Meta-regression (*P*-value)
				*I* ^2^ (%)	*P*-value	
Hereditary Rb	6	3639	17.55(13.10, 23.51)	91.5	<0.001	–
Type of SPCs in hereditary Rb						0.028
Soft tissue sarcoma	8	5079	202.93(114.10, 360.93)	94.9	<0.001	
Bone tumor	5	4147	442.91(191.63, 1023.68)	97.5	<0.001	
Central nervous system (CNS)	5	3341	12.82(8.73, 18.82)	2.0	0.395	
Female breast cancer	4	3195	3.68(2.52, 5.37)	0.0	0.876	
Nasal cavity tumor	2	2091	591.06(162.79, 2146.01)	91.9	<0.001	
Treatment techniques in hereditary Rb						–
Radiation therapy (RT)	2	1869	162.90(95.94, 276.58)	86.4	0.007	
Chemotherapy therapy (CT)	1	963	236.00(161.10, 339.41)	–	–	
RT plus CT	1	906	145.10(107.50, 195.86)	–	–	
Nonhereditary Rb	5	2524	1.36(0.90, 2.04)	55.1	0.064	–
Type of SPCs in nonhereditary Rb						0.003
Central nervous system (CNS)	3	1231	4.62(1.41, 15.15)	0.0	0.496	
Soft tissue sarcoma	2	560	22.31(9.45, 52.63)	0.0	0.964	
Female breast cancer	3	1932	1.72(0.90, 3.30)	22.0	0.278	

### Type of SPCs in hereditary Rb

**Figure 3:**
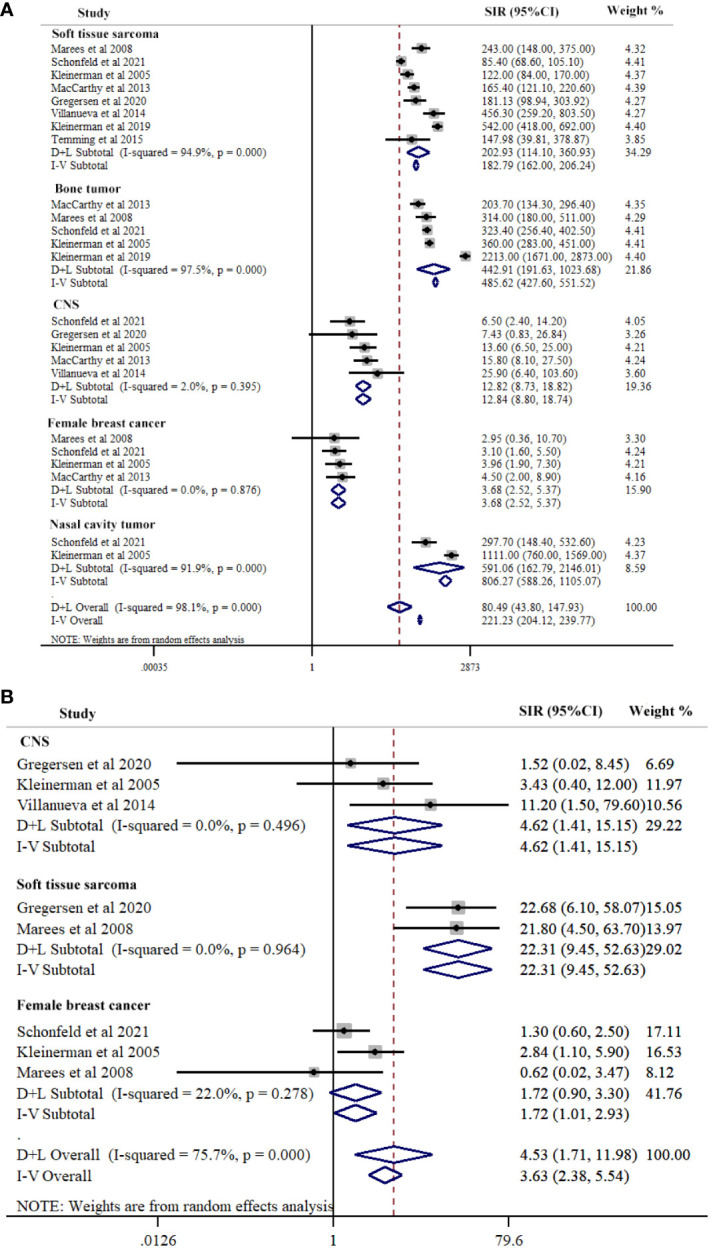
Subgroup-analysis of Risk for second primary cancers types among hereditary **(A)** and nonhereditary **(B)** retinoblastoma patients. Subtotal effects were estimated using random effects model (D+L Subtotal).

Our study conducted a subgroup analysis according to the types of SPC in hereditary Rb, including soft tissue sarcoma, nasal cavity cancer, CNS cancer, bone cancer, and female breast cancer. The summary estimate of SIR for nasal cavity tumor was the highest (SIR=591.06, 95%CI=162.79-2146.01; *I* ^2^ = 91.9%, *P*<0.001) across of the five cancer types, followed by bone tumor (SIR=442.91, 95% CI=191.63-1023.68; *I* ^2^ = 97.5%, *P*<0.001), soft tissue sarcoma (SIR=202.93, 95% CI=114.10-360.93; *I* ^2^ = 94.9%, *P*<0.001), CNS (SIR=12.84, 95%CI=8.80-18.74; *I* ^2^ = 2.0%, *P*=0.395), and female breast cancer (SIR=3.68, 95%CI=2.52-5.37; *I* ^2^ = 0.0%, *P*=0.876). Meta-regression results indicated significant differences between the five cancer types (*P*=0.028) (**Figure 3A**; **Table 2**).

### Type of SPCs in nonhereditary Rb

SPCs type contained soft tissue sarcoma, CNS, and female breast cancer in nonhereditary Rb. The summary estimate of SIR for soft tissue sarcoma was the highest (SIR=23.31, 95% CI=9.46-52.63; *I* ^2^ = 0.0%, *P*=0.964) across the three cancer sites, followed by CNS (SIR=4.62, 95% CI=1.41-15.15; *I* ^2^ = 0.0%, *P*=0.496), and female breast cancer (SIR=1.72, 95% CI=1.01-2.93; *I* ^2^ = 22.0%, *P*=0.278). Meta-regression results indicated significant differences between the three cancer sites (*P*=0.003) (**Figure 3B**; **Table 2**).

### Treatment techniques in hereditary Rb

**Figure 4:**
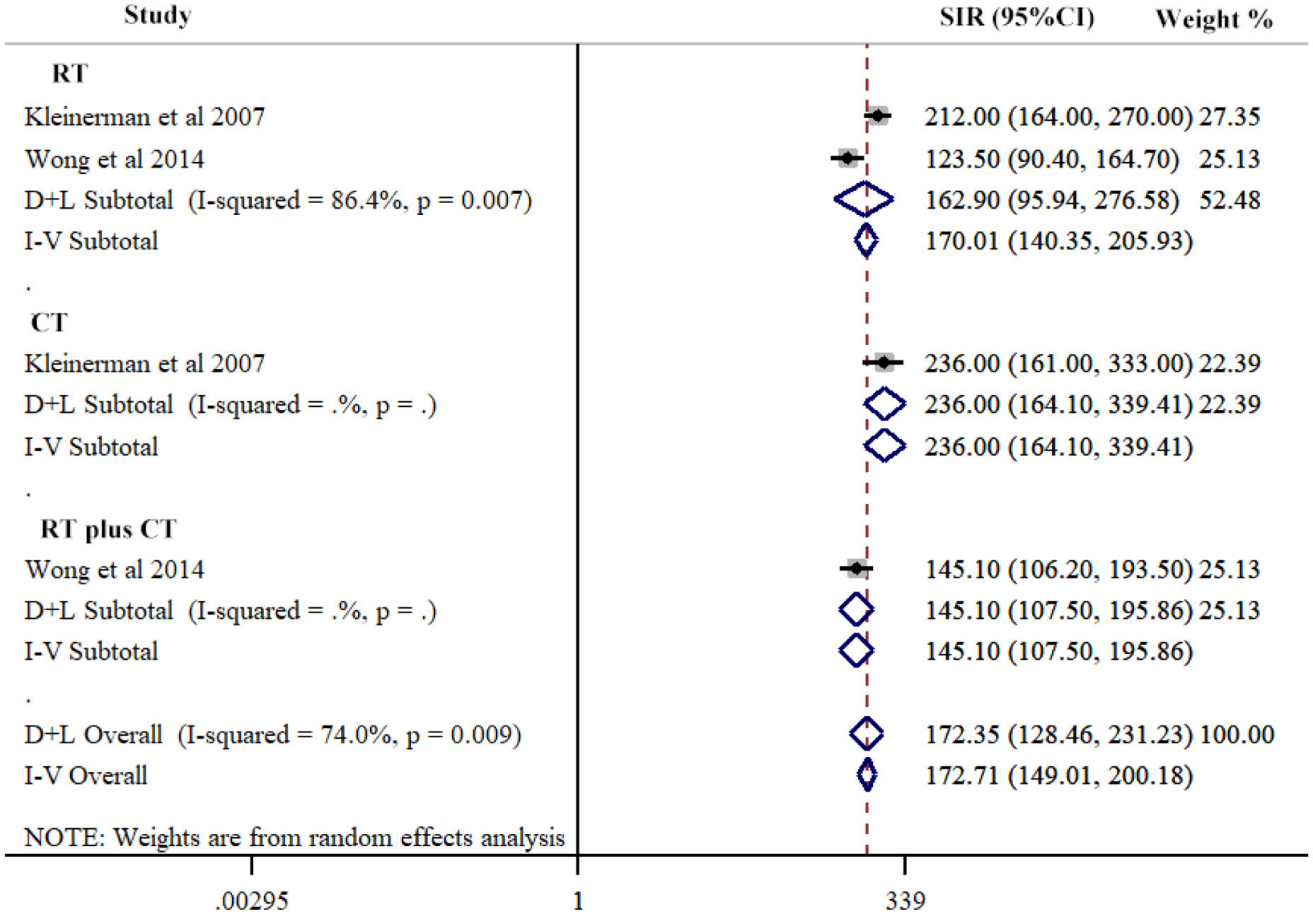
Risk of second primary cancers overall among hereditary retinoblastoma patients by different treatment techniques. Subtotal effects were estimated using random effects model (D+L Subtotal).

Our study conducted a subgroup analysis according to the treatment techniques in hereditary Rb, including RT, CT, and RT plus CT. The three groups had comparable SIRs of SPCs: RT, (SIR=162.90, 95% CI=95.94-276.58; *I* ^2^ = 86.4%, *P*=0.007); CT, (SIR=236.00, 95% CI=164.10-339.14); and RT plus CT, (SIR=141.10, 95% CI=107.50-195.86) (**Figure 4**; **Table 2**).

### Publication bias

**Figure 5:**
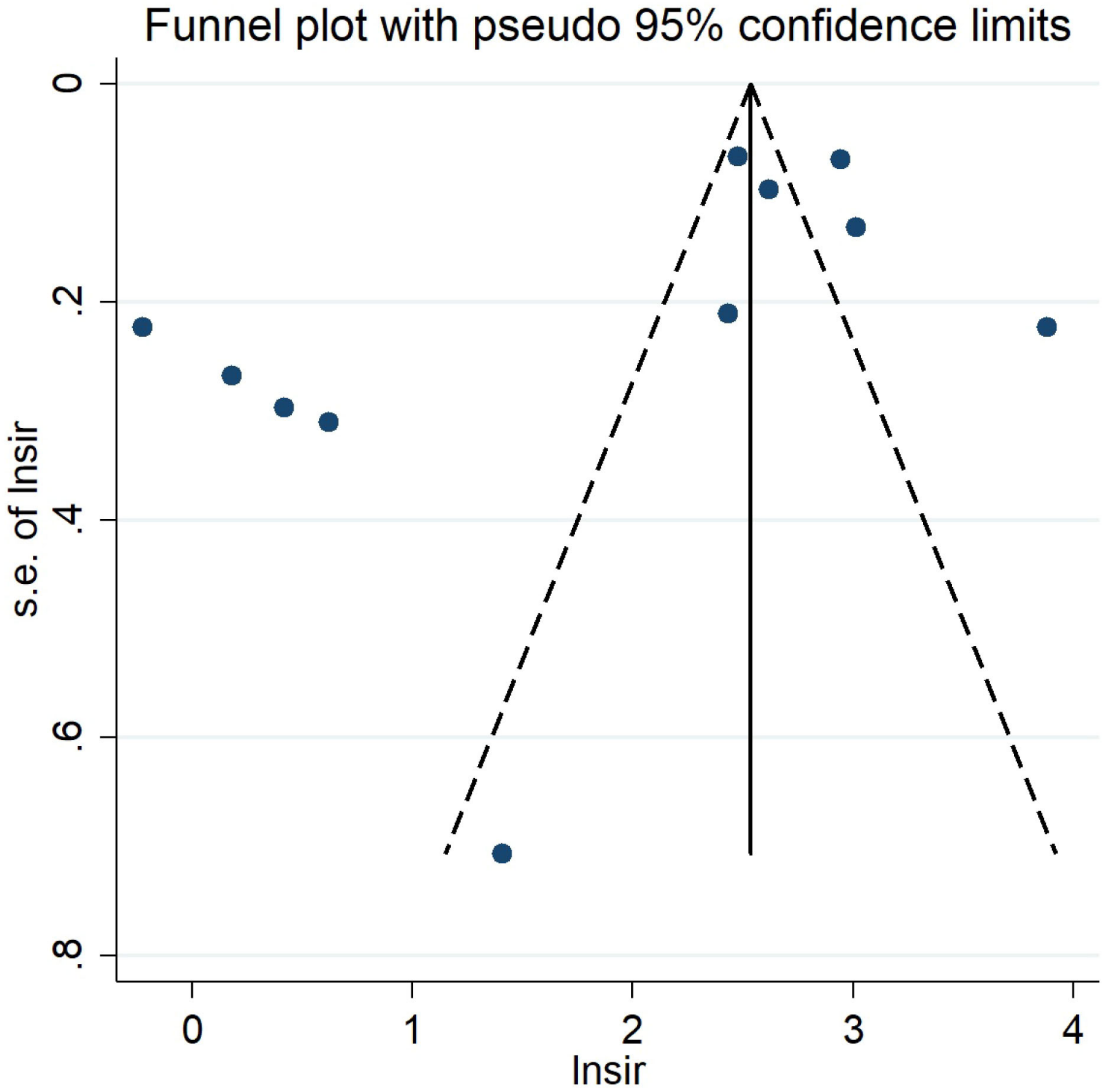
Funnel plot for second primary cancers overall among hereditary and nonhereditary retinoblastoma patients.

Visual inspection of the funnel plot indicated some publication bias while Egger’s test suggested no publication bias (*P*=0.074). Funnel plot is presented in **Figure 5**.

## Discussion

This study was the first meta-analysis to assess the incidence for developing SPCs after hereditary and nonhereditary Rb, as well as the risk for the subtypes of SPCs. Compared to the general population, the risk of developing SPCs was 17-fold higher in hereditary Rb survivors, while nonhereditary Rb survivors did not have a significantly elevated risk. Past studies have also demonstrated the significantly elevated risk of SPCs in hereditary R (12, 14, 21, 24, 26–28). Temming et al. (2017) reported that SPCs remained a significant concern in heritable Rb patients (10). Additionally, Schonfeld et al. (2021) demonstrated that hereditary Rb patients had a statistically significant elevated risk of SPCs, while SPCs risk was not increased after nonhereditary Rb (12). The oncogenic *RB1* alteration and compromised tumor suppressor function contributed to the increased risk of SPCs in hereditary Rb survivors (14, 23). The present review included a range of studies from different regions and a large number of participants, and our finding emphasize the importance of SPC screening in hereditary Rb survivors, while non-hereditary Rb survivors might not require additional cancer screening due to the low risk.

Hereditary Rb survivors had significant risks of soft tissue sarcomas, cancers of the nasal cavities, CNS, bone tumors, and female breast cancer, which is consistent with previous findings (27, 29). The highest SIR was for nasal cavity tumor (591-fold), followed by bone tumor (442-fold), soft tissue sarcoma (202-fold), CNS tumor (12-fold), and female breast cancer (3-fold). Kleinerman et al. (2005) stated that the doses to the nasal region were high (3.2 to 34 Gy) in their series, which might explain the increased risk for nasal cavity tumors among hereditary Rb survivors (21). Furthermore, Rb pathway alterations have been identified in various soft tissue sarcomas, even in patients without a history of Rb (30). One study found that hereditary Rb patients treated with surgery alone had an elevated risk of breast cancer, indicating the importance of Rb hereditary for breast cancer (29). We did not observe an increased risk for other epithelial malignancies, including kidney, bladder, uterus, pancreas or lung.

Nonhereditary Rb patients did not have a statistically significant increased risk of SPCs overall compared to the general population. Nonetheless, there is a statistically significant risk of soft tissue sarcomas (23-fold), CNS (4-fold), and female breast cancer (1.7-fold). Similarly, Marees et al. (2008) found a statistically significantly elevated risk of soft tissue sarcomas (21-fold) among nonhereditary patients (24). The occurrence of SPCs may be a radiation effect among nonhereditary patients. SPC screening programs should be focused the types of SPCs with the highest risks for the maximized benefites and minimized risks.

Regarding the treatments of Rb, chemotherapy and RT increased the risk of SPCs among hereditary Rb patients. EBRT is one of the crucial risk factors of SPCs in hereditary Rb patients, with or without systemic chemotherapy. Studies demonstrated a higher incidence of SPCs when both modalities were used together (31, 32). Other studies indicated that chemotherapy was an independent risk factor for SPCs in Rb patients, especially sarcomas (10, 19). Systemic chemotherapy replaced radiotherapy for Rb since the mid-1990s; however, EBRT is still frequently used in advanced diseases in low-income countries (13). Retrospective studies in this review included patient data from a long period of time (i.e., the year 1914-2016), and the treatment techniques have changed drastically, especially in developed countries. Since then, international guidelines have been established and continually updated to help clinicians estimate risks from radiation exposure doses, and further precautions are taken to protect the cornea, lens, and surrounding structures (33). RT has been discarded for patients with bilateral Rb due to the increased risk of SPCs. Following the decline of RT, a consensus emerged among partitioners to utilize multi-agent systemic chemotherapy for bilateral cases. However, our meta-analysis found that, among hereditary Rb survivors, the risks of different therapies (i.e., RT alone, chemotherapy alone, and RT in combination with chemotherapy) were comparable. The result is contradictory to the previous claim that chemotherapy might serve a role of protective factor for the development of SPCs (6). This finding highlighted the need for SPC surveillance even in the declined use of radiotherapy, and innovative treatments with reduced SPC rates should be investigated in future clinical trials. Nonetheless, study findings should be interpreted cautiously with regard to the current treatment protocol of different regions.

Considering the elevated risk among hereditary Rb patients, life-long surveillance for SPCs is important in this population. Patient education and regular screening of SPCs may enhance patients’ survivorship and survival. Although nonhereditary Rb patients did not have an elevated overall risk of SPCs, soft tissue sarcoma should be screened and awareness of SPCs should be improved. EBRT remains one of the crucial risk factors of SPCs. Therefore, the physician should pay attention to radiation protection for Rb patients. It is also essential to consider the risks of secondary cancers in comparison to other radiation treatment modalities to have more helpful information when patients with cancer undergo radiation therapy more than once. Surveillance for SPCs is important, considering the significantly increased risk in survivors with hereditary Rb. Yearly oncologic checkup is routine in a few Rb centers and regular whole-body magnetic resonance imaging has been suggested (14). However, the effects and potential risks (i.e., cost and psychological distress) of the SPC screening should require further evaluation.

This review has several limitations. First, all included studies in our meta-analysis were retrospective designs, which could lead to recall bias. Second, of the ten included studies, nine were conducted in high-income countries. As treatment techniques and quality of service might vary greatly in different countries, the study findings might not be generalizable to developing countries. Further studies should be conducted in low and middle-income countries. Third, this study only analyzed five SPCs, while other types were not considered due to insufficient data. Fourth, the heterogeneity in both SIRs of SPCs in hereditary and nonhereditary Rb was substantial. While the heterogeneity in nonhereditary Rb was explained by SPC types, the heterogeneity in heterogeneity in hereditary Rb cannot be explained by SPC types or treatment techniques. The heterogeneity might be due to the different subtypes in hereditary Rb patients. Dommering et al. (2021) found that adjusting for age and therapy, there was a higher risk of SPCs for Rb patients carrying a recurrent nonsense mutation, but a reduced risks for patients with a low penetrance mutation (11). Because the included studies did not further divide hereditary Rb into genotype-phenotype subtypes, the heterogeneity cannot be investigated through statistical analysis. Furthermore, misclassification of Rb patients could occur in the absence of genetic testing (i.e., before 1966) (14). Future studies should take full advantage of genetic testing and divide patients into more accurate subgroup genotype groups, and the findings can further inform SPC surveillance strategies in this patient population. Other possible causes of heterogeneity include years of patients investigated, because the treatment techniques have changed drastically over the past decades. The heterogeneity might also be caused by the diverse medical systems included in the study: treatment plans and medical resources can vary greatly from country to country. Future studies should clearly report the relevant information in their manuscripts and future review studies should further investigate the heterogeneity with added information.

The findings of our study suggested that patients with hereditary Rb had a significantly elevated risk for SPCs, while those with nonhereditary Rb did not. Significant differences were found between different SPCs types. Hereditary Rb survivors had the highest SPC risk for nasal cavity tumor, followed by bone tumor, soft tissue sarcoma, CNS, and female breast cancer. Nonhereditary Rb survivors had the highest SPC risk for soft tissue sarcoma, followed by CNS and female breast cancer. RT alone, chemotherapy alone, and RT in combination with chemotherapy all significantly increased the risk of SPCs. Therefore, the physician should pay attention to radiation protection for Rb patients, especially hereditary patients and patients receiving RT and chemotherapy. Surveillance for SPCs is important in hereditary Rb survivors, considering the significantly increased risk of SPCs. Future studies should investigate the feasibility and effectiveness of SPC surveillance program in Rb patients.

## Scope statement

This systematic review and meta-analysis aim to investigate the risk of second primary cancers (SPCs) among patients with retinoblastoma (Rb). By synthesizing data from multiple studies, the study assesses the standardized incidence ratios (SIRs) of SPCs in both hereditary and nonhereditary Rb patients.

The findings provide insights into the long-term risk of SPCs in Rb patients, with a focus on identifying any differences between hereditary and nonhereditary cases. Furthermore, the study examines the impact of treatment modalities, such as chemotherapy and radiation therapy, on the risk of SPCs.

The research has direct clinical relevance, as it informs healthcare professionals about the increased risk of SPCs in hereditary Rb patients and the potential influence of treatment techniques. The findings may have implications for treatment decision-making, follow-up care, and radiation protection measures in the management of Rb patients.

Given its focus on oncology and the importance of understanding the risk of SPCs in Rb patients, this manuscript aligns well with the scope of Frontiers in Oncology. The study’s comprehensive approach, including the systematic review and meta-analysis, contributes to the existing knowledge base and may inform future research and clinical practices related to retinoblastoma and second primary cancers.

## Data availability statement

The original contributions presented in the study are included in the article/supplementary material. Further inquiries can be directed to the corresponding author.

## Author contributions

JS: Writing – review & editing, Writing – original draft, Conceptualization. XG: Writing – original draft, Conceptualization. LW: Writing – review & editing, Formal analysis.
